# Bronchial myeloid sarcoma secondary to acute monoblastic leukemia (AML-M5): a rare Case Report

**DOI:** 10.3389/fmed.2025.1703260

**Published:** 2025-12-01

**Authors:** Zhen Yang, Yuzhi Lu, Shenglan Ye, Jixiang Ni, Hongling Hu

**Affiliations:** Department of Respiratory and Critical Care Medicine, The Central Hospital of Wuhan, Tongji Medical College, Huazhong University of Science and Technology, Wuhan, Hubei, China

**Keywords:** myeloid sarcoma, acute monoblastic leukemia, endotracheal neoplasm, immunohistochemistry, case report

## Abstract

Bronchial myeloid sarcoma (BMS) is an extramedullary rare extramedullary manifestation of acute myeloid leukemia (AML), with only eight cases previously reported worldwide. In this report, we present the ninth documented case of BMS occurring in a 47-years-old man with acute monoblastic leukemia (AML-M5) during venetoclax therapy. The patient initially presented with cough and fever, leading to a misdiagnosis of bronchopneumonia. However, following a biopsy of the endobronchial lesion obtained via bronchoscopy and subsequent immunohistochemical analysis, a diagnosis of BMS was made. Following suboptimal response to venetoclax, a sequential therapeutic approach was initiated, involving salvage chemotherapy (liposomal mitoxantrone and cytarabine), consolidation with azacitidine and venetoclax, and subsequent allogeneic hematopoietic stem cell transplantation (HSCT). This approach resulted in complete remission. Short-term follow-up demonstrated sustained disease-free survival, with restored bronchial patency and normalized hematological parameters. This case underscores the potential for BMS to arise during novel targeted therapies and highlights the efficacy of a multimodal treatment strategy combining sequential chemotherapy and HSCT. Early diagnostic suspicion in patients with AML presenting with pulmonary symptoms, along with comprehensive immunohistopathological evaluation, is critical for effective management of this rare condition.

## 1 Introduction

Myeloid sarcoma (MS) is an uncommon, aggressive form of blood cancer characterized by a tumor mass composed of immature myeloid cells that forms outside the bone marrow, disrupting normal tissue structure (1). First described by Burns in 1811, MS is a rare neoplastic lesion characterized by immature cells (2). It is also known as chloroma, granulocytic sarcoma, myeloblastoma, myelosarcoma, and extramedullary acute myeloid leukemia (AML) (3). MS can develop in various organs, most frequently in the bone, adjacent soft tissues, and lymph nodes, although it may occur anywhere (4). However, bronchial involvement is exceedingly rare, with only eight reported cases worldwide (5–12).

In the present report, we describe a case of a patient with acute monoblastic leukemia (AML-M5) receiving venetoclax-based therapy who presented with recurrent cough and fever. The patient was initially diagnosed with bronchogenic pneumonia and later confirmed to have bronchial myeloid sarcoma (BMS). Given the limited number of reports on MS-related bronchial involvement, this case represents a meaningful contribution to the existing medical literature.

## 2 Case presentation

A 47-years-old male was admitted to the hospital on February 21, 2025, with a 2-months history of cough and fever. The patient was diagnosed with AML-M5 in December 2024 and had been receiving venetoclax-based therapy since January 15, 2025, according to a standard dose-escalation regimen (100 mg daily on day 1, 200 mg daily on day 2, and 400 mg daily on days 3–10). Owing to intolerance, the dose was subsequently reduced to 200 mg daily (days 11–28). However, owing to persistent symptoms and inadequate disease control, the patient was re-admitted.

On physical examination, his vital signs were as follows: body temperature of 36.4 °C, pulse of 88 beats/min and regular, respiratory rate of 17 breaths/min and regular, blood pressure of 102/64 mmHg, and SpO2 98% on room air. While the patient was conscious, he appeared anemic. No superficial lymphadenopathy was detected. Coarse breath sounds were noted in both lungs, with moist rales auscultated. Cardiac examination revealed a regular rhythm without murmurs. The abdomen was soft and non-tender, with no hepatosplenomegaly. No edema was observed in the lower extremities.

Laboratory investigations revealed severe pancytopenia, with the following parameters: leukocyte count, 0.38 × 10^9^/L (reference range: 3.5–9.5 × 10^9^/L); hemoglobin, 59 g/L (reference range: 130–175 g/L); and platelet count, 3.0 × 10^9^/L (reference range: 125–350 × 10^9^/L). C-reactive protein (CRP), an inflammatory marker, was elevated at 54.95 mg/L (reference range: 0–6 mg/L). Moreover, electrolyte and protein metabolism abnormalities were noted, including hypocalcemia (calcium 2.09 mmol/L; reference range: 2.2–2.7 mmol/L), hypomagnesemia (magnesium 0.73 mmol/L; reference range: 0.77–1.03 mmol/L), hypoproteinemia (total protein 53.5 g/L; reference range: 65–85 g/L), and hypoalbuminemia (albumin 34.7 g/L; reference range: 40–55 g/L). Coagulation studies revealed an elevated D-dimer level of 1.41 μg/mL FEU (reference range: 0–0.55 μg/mL). Procalcitonin and routine coagulation parameters were within normal limits.

**FIGURE 1:**
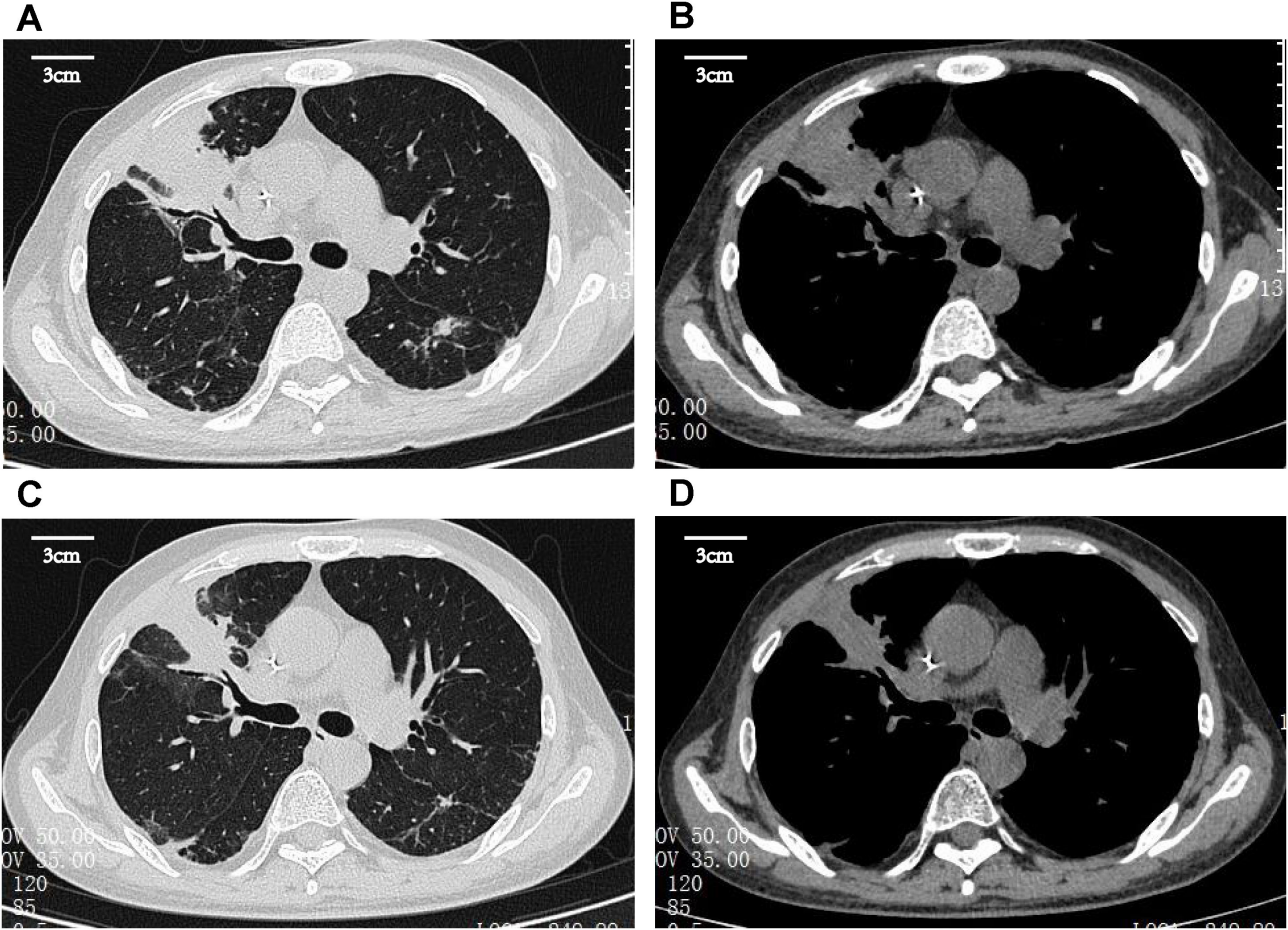
Chest computed tomography (CT) images. **(A,B)** February 22, 2025, CT scans of the chest indicated a right upper lung consolidation measuring 4.6 cm × 2.5 cm, with an irregular shape and ill-defined borders, suspicious for right upper lobe airway obstruction. **(C,D)** July 1, 2025, follow-up pulmonary CT imaging indicated reduced absorption (approximately 50%) of the consolidation in the right upper lobe compared to previous imaging.

**FIGURE 2:**
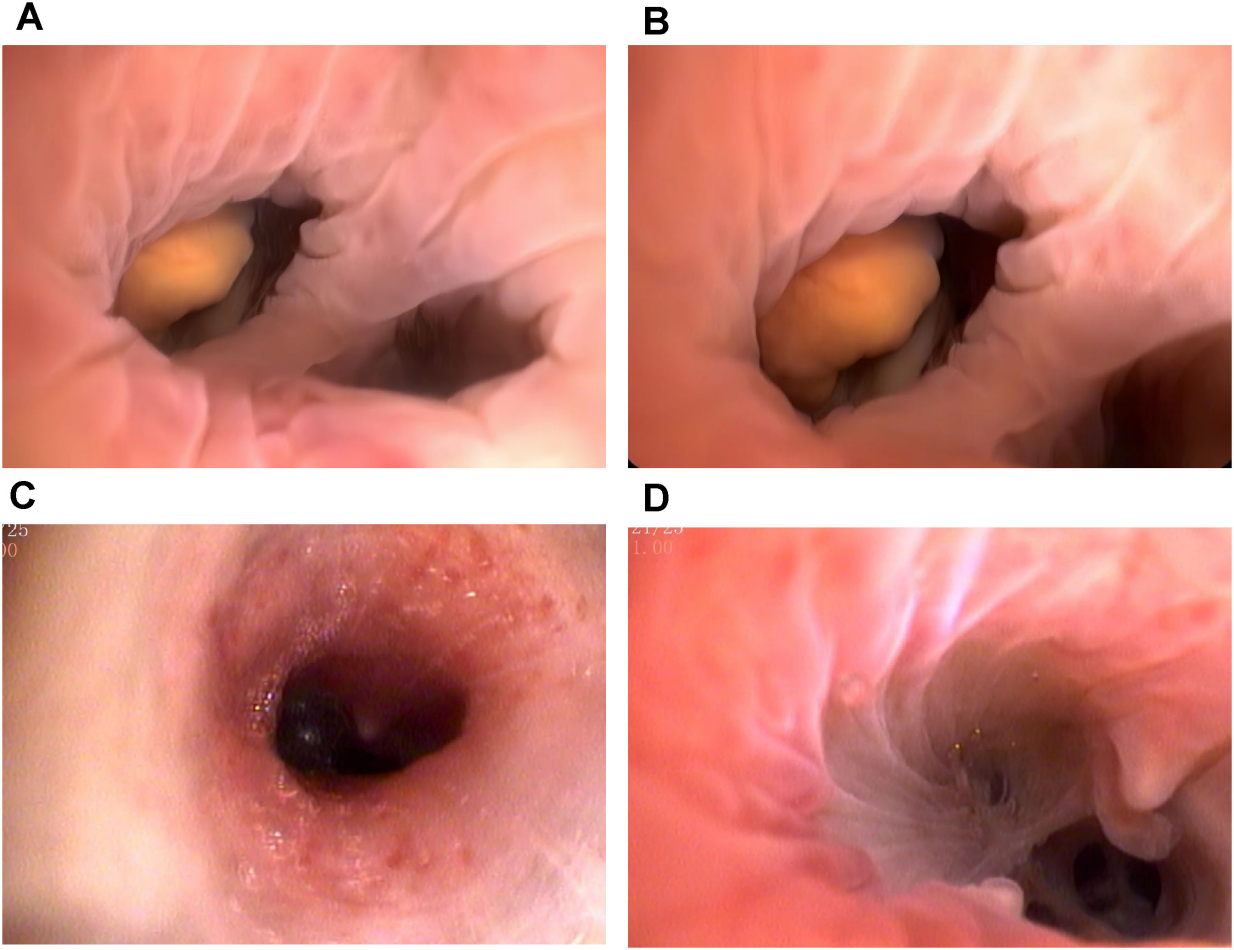
Bronchoscopic images before and after the treatment. **(A,B)** The patient underwent bronchoscopy and identified a yellowish, endotracheal neoplasm at the right upper lobe anterior segment orifice. The friable mass demonstrated focal hyperemia and surface irregularity, bled readily upon contact, and nearly completely occluded the bronchial lumen. **(C,D)** No abnormalities were found on re-examination via bronchoscopy after treatment, but the interval between the branches was broken.

**FIGURE 3:**
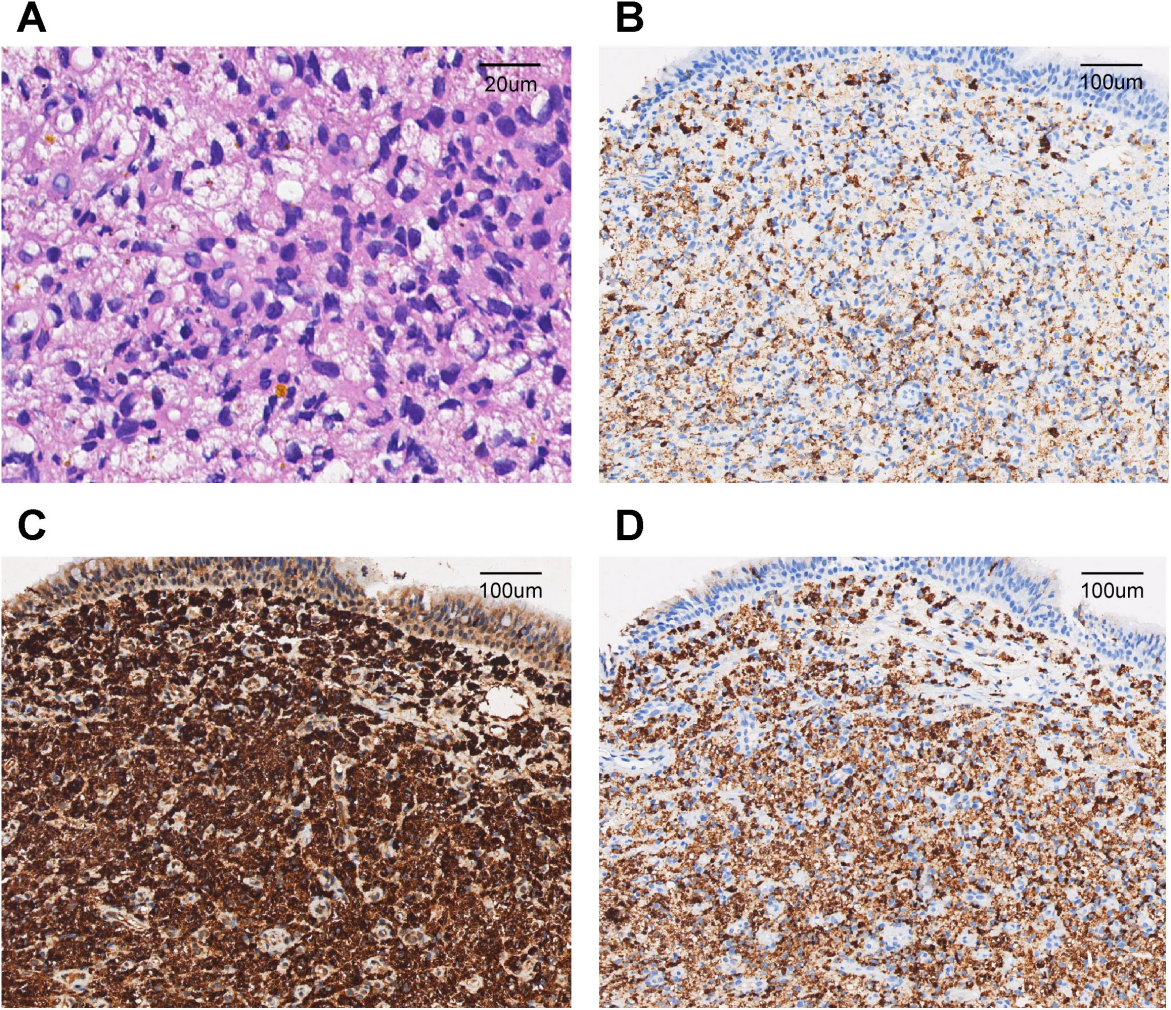
Pathological findings of the endobronchial biopsy. **(A)** Diffuse infiltration of bronchial mucosa by medium to large immature myeloid cells (hematoxylin and eosin staining, ×200). **(B)** Immunochemistry showed positive staining for myeloperoxidase (MPO) in neoplastic cells (×40). **(C)** Immunochemistry showed positive staining for CD68 in neoplastic cells (×40). **(D)** Immunochemistry showed positive staining for Lysozyme in neoplastic cells (×40).

Computed tomography (CT) of the chest revealed a right upper lung consolidation measuring 4.6 cm × 2.5 cm, with an irregular shape and ill-defined borders, suspicious for right upper lobe airway obstruction. No pleural effusion or mediastinal lymph node enlargement was observed (Figures 1A, B). Bronchoscopy revealed a yellowish, nodular, endotracheal neoplasm at the orifice of the anterior segment of the right upper lobe bronchus. The lesion had a generally smooth surface with areas of local hyperemia and roughness, was friable, and bled easily on contact. It nearly completely obstructed the anterior segmental bronchial lumen, leaving only a narrow slit for ventilation, and the surrounding mucosa was edematous (Figures 2A, B). Biopsy forceps were used to collect endobronchial biopsies from the specified area. Histological examination revealed bronchial mucosa with a diffuse infiltrate of immature myeloid cells of medium and large size (Figure 3A). Immunohistochemistry confirmed their myeloid origin, revealing exhibited strong and diffuse positivity for CD13 (∼90% of cells), CD33 (∼95%), CD43 (∼90%), and lysozyme (∼85%); focal, moderate-to-strong positivity for CD68 (∼40%) and CD163 (∼35%); and focal, moderate positivity for myeloperoxidase (∼30%). The proliferation index Ki-67 was approximately 5%. In contrast, markers for lymphoid lineage (CD3 and CD20), epithelial origin (Pan-CK), and hematopoietic stem/progenitor cells (CD34 and CD117) were all negative (Figures 3B–D). A concurrent bone marrow aspirate revealed that the monocytic series still accounted for 30% of nucleated cells, with primitive and immature monocytes accounting 5%, indicating failure to achieve remission from leukemia.

Given the suboptimal response to venetoclax, salvage chemotherapy with mitoxantrone hydrochloride liposome injection in combination with cytarabine (the MA regimen) was initiated on March 10, 2025. This regimen consisted of liposomal mitoxantrone (30 mg on day 1) and cytarabine (150 mg daily on days 1–7). A subsequent bone marrow assessment revealed severe hypoplasia, characterized by markedly hypocellular marrow with a relative increase in lymphocytes (98%) and no identifiable myeloblasts. To consolidate the response, therapy with azacitidine (100 mg daily on days 1–7) combined with venetoclax (using the standard dose-escalation regimen: 100 mg daily on day 1, 200 mg daily on day 2, and 400 mg daily on days 3–28) was initiated on May 27, 2025. By July 2025, follow-up evaluations indicated bone marrow recovery. Specifically, the indicated marrow was markedly hypercellular, with a significant increase in erythroid precursors and no morphologically identifiable myeloblasts. Flow cytometry detected no minimal residual disease, and peripheral blood counts had normalized. Concurrent CT imaging indicated reduced absorption (approximately 50%) of consolidation in the right upper lobe compared to previous imaging (Figures 1C, D). Bronchoscopic evaluation revealed resolution of endotracheal neoplasm in the same lobe, with patency of the lumen restored. The carina of the anterior segment of the right upper lobe bronchus exhibited mild widening and fusion, suggesting architectural remodeling post-infiltration; however, no residual or recurrent neoplastic lesions were observed (Figures 2C, D).

Following confirmation of complete remission, the patient underwent allogeneic hematopoietic stem cell transplantation (HSCT) at an external specialized center in August 2025. Post-transplant monitoring followed a standardized protocol: weekly to twice weekly for the first 6 months, monthly from 6 months to 1 year, every 3 months during the second year, and every 6 months thereafter. Although specific follow-up data from the external center were unavailable, a telephone follow-up in October 2025 confirmed that the patient remained asymptomatic with persistently normal blood counts. The final diagnosis of BMS was confirmed based on histology, immunohistochemistry, bronchoscopy, and chest CT findings, and was consistent with secondary BMS arising from AML-M5.

## 3 Discussion

Myeloid sarcoma is a rare clinical entity characterized by a solid mass of immature myeloid cells. This condition typically occurs in patients with a history of myeloproliferative neoplasm, myelodysplastic syndrome, or chronic myeloid leukemia. The overall incidence rate of MS is low, estimated at 2 per 100,000 adults and 0.7 per 100,000 children (13). It is most frequently associated with AML, affecting approximately 2.5%–9.1% of patients with AML (1), similar to the case discussed here. MS often presents as an early manifestation or complication of AML and is particularly associated with the AML-M5 subtype, indicating enhanced disease aggressiveness and poor prognosis. In this report, we describe the ninth documented case globally of BMS secondary to AML-M5 that emerged during venetoclax therapy. The patient achieved complete remission through salvage chemotherapy and allogeneic HSCT, maintained during short-term follow-up. This case report enables systematic comparison with eight previously published cases.

Our patient developed BMS 2 months following the initial AML-M5 diagnosis, during a period of suboptimal venetoclax dosing. BMS typically manifests in patients with established AML, either at initial presentation, during remission, or at relapse. For example, Dugdale et al. (5) and Faiz et al. (6) described BMS in patients with active AML, whereas Genet et al. (7) and Psathakis et al. (8) reported cases of isolated bronchial recurrence following initial remission. The distinctive feature of our case lies in the development of BMS during targeted therapy with venetoclax, a BCL-2 inhibitor. Notably, BMS development is rarely associated with extramedullary progression, underscoring the potential for MS development even in the context of novel molecular therapies.

The clinical presentation of BMS is diverse and non-specific, frequently leading to diagnostic delays. Our patient presented with cough and fever, consistent with descriptions by Wang et al. (9) and Vennepureddy et al. (10), although hemoptysis, which was reported in the latter case, was absent. Unlike the case reported by Dugdale et al. (5), our patient did not develop respiratory failure. Imaging findings revealed bronchial obstruction with consolidation, aligning with reports by Faiz et al. (6) and Stafford et al. (11); however, our patient did not exhibit the extensive mediastinal involvement described by Psathakis et al. (8). In our case, the initial misdiagnosis of bronchopneumonia, which was based on clinical presentation, CT imaging, and elevated CRP levels, highlights the diagnostic challenges inherent to BMS. These included non-specific clinical and radiographic features mimicking common pulmonary conditions, low clinical suspicion due to disease rarity, and reliance on invasive bronchoscopic procedures for definitive diagnosis. Therefore, heightened vigilance is warranted in patients with AML presenting with new pulmonary symptoms or lesions.

Histopathological examination of biopsy specimens remains the diagnostic gold standard for BMS. In our case, bronchoscopy revealed yellowish, nodular, friable lesions, consistent with the “pinkish-white” or “greenish” submucosal infiltrates described by Stafford et al. (11) and Psathakis et al. (8). Our immunohistochemical analysis demonstrated strong positivity for CD13, CD33, CD43, and lysozyme, with focal expression of MPO and CD68, confirming monoblastic differentiation. The absence of epithelial markers (Pan-CK) effectively excluded carcinoma, while negative lymphoid markers (CD3, CD20, and TdT) ruled out lymphoproliferative disorders. A low Ki-67 proliferation index further helped distinguish BMS from highly aggressive small round cell tumors or lymphomas. This immunoprofile corresponds with findings reported by Vennepureddy et al. (10) and Wang et al. (9). Notably, the absence of CD34 and CD117, also observed in other MS cases such as that described by Podgaetz et al. (12), may reflect phenotypic divergence between medullary and extramedullary disease.

To date, no consensus exists regarding optimal BMS management. Therapeutic strategies for MS should consider multiple factors, including tumor location and size, relationship to surrounding structures, and patient age and performance status (14). As MS represents an extramedullary manifestation of AML, treatment generally follows AML principles, prioritizing systemic chemotherapy while considering local therapies (radiotherapy/surgery) as adjuncts (15). Localized approaches alone are insufficient for disease eradication, necessitating systemic chemotherapy to control extramedullary lesions and prevent systemic relapse (16). Following a suboptimal response to venetoclax treatment, our patient received salvage the MA regimen, followed by consolidation with azacitidine and venetoclax, and ultimately successful allogeneic HSCT. This therapeutic sequence was selected based on several considerations. First, compared to conventional anthracycline-based regimens demonstrating limited efficacy in earlier reports (5, 7), liposomal mitoxantrone offers superior pharmacokinetics and reduced cardiotoxicity, making it particularly advantageous for patients requiring intensive therapy. Second, the sequential treatment strategy aimed to maintain remission while reducing pre-transplantation toxicity. Notably, the outcomes of the individualized approach contrast with the poor outcomes in earlier cases (5, 7). Specifically, while the cases recently reported by Wang et al. (9) and Podgaetz et al. (12) also achieved remission through chemotherapy, only the latter experienced long-term survival post-HSCT. Allogeneic HSCT plays a pivotal role in achieving long-term disease control. Patients with MS undergoing HSCT achieve 5-years overall and leukemia-free survival rates of 48% and 36%, respectively (10). Our patient met ideal transplantation criteria, including young age, adequate organ function, and sensitivity to salvage chemotherapy. Moreover, patients with patients undergoing transplantation reportedly exhibit 1- and 3-years overall survival rates of 71.4% and 64.3%, respectively (17). Another study demonstrated 1- and 3-years cumulative overall survival rates of 64.9% and 48.6% in patients with AML-M5 undergoing allogeneic HSCT (18). Consistently, the pre-transplantation disease status significantly influences out comes, with optimal results achieved when the transplant is performed during complete remission (18).

The prognosis of BMS remains generally poor, with median survival maybe under 12 months (8, 11). Dugdale et al. (5) observed rapid clinical deterioration and death within 5 days of cytarabine and vincristine treatment. In contrast, Wang et al. (9) reported the first BMS case treated with the HAG regimen (homoharringtonine, cytarabine, and G-CSF), achieving complete radiological resolution after three cycles and no recurrence during 1-year follow-up. In another exploratory case (8), a patient with relapsed AML refractory to salvage chemotherapy received oral lenalidomide as palliative treatment, resulting in the resolution of abdominal lesions and partial thoracic response. However, the patient ultimately succumbed to sepsis and disease progression 12 months after diagnosis. Conversely, our patient achieved complete remission and remained disease-free during short-term follow-up after HSCT. In addition, prognostic analysis identifies several key factors influencing BMS outcomes. Our case exhibited favorable characteristics, including relatively young age (47 years), absence of significant comorbidities, and limited disease extent without mediastinal involvement, in contrast to the poorer outcomes typically observed in older patients with extensive disease (8, 10). Consistent with previous studies (9, 12), early diagnosis through prompt bronchoscopic evaluation and rapid treatment initiation likely contributed to the successful outcome in our case, emphasizing the importance of timely intervention.

This report has some limitations. First, as a single-center case report, our study cannot account for population heterogeneity, limiting the generalizability of our findings. Second, as our patient received a multimodal treatment regimen, it is difficult to distinguish the contribution of individual components within the combined treatment regimen. Finally, the follow-up period was short, and long-term efficacy and durability of response remain unclear. Therefore, future multi-center collaborations establishing case registries and prospective studies are necessary to determine optimal management strategies.

## 4 Conclusion

As the ninth reported BMS case, this report demonstrates the potential for achieving favorable outcomes through systematic diagnosis, sequential chemotherapy, and well-timed transplantation in this rare disease. While the results are encouraging, larger, multi-center studies are essential to validate these findings and establish evidence-based guidelines for BMS management.

## Data Availability

The raw data supporting the conclusions of this article will be made available by the authors, without undue reservation.
